# Solid state treatment with *Lactobacillus paracasei* subsp. *paracasei* BGHN14 and *Lactobacillus rhamnosus* BGT10 improves nutrient bioavailability in granular fish feed

**DOI:** 10.1371/journal.pone.0219558

**Published:** 2019-07-11

**Authors:** Jovanka Lukic, Goran Vukotic, Nemanja Stanisavljevic, Dejana Kosanovic, Zsuzsanna Molnar, Jelena Begovic, Amarela Terzic-Vidojevic, Galina Jeney, Uros Ljubobratovic

**Affiliations:** 1 Institute of Molecular Genetics and Genetic Engineering (IMGGE), University of Belgrade, Belgrade, Serbia; 2 Faculty of Biology, University of Belgrade, Belgrade, Serbia; 3 Institute of Virology, Vaccines and Sera “Torlak”, Belgrade, Serbia; 4 Research Institute for Fisheries and Aquaculture (NAIK HAKI), Szarvas, Hungary; University of Illinois, UNITED STATES

## Abstract

The aim of this research was to improve nutritive value of fishmeal-based feed by lactobacilli in order to achieve satisfactory nutrient availability needed to support fish development. Feed was solid-state treated at a laboratory scale with the combination of *Lactobacillus paracasei* subsp. *paracasei* BGHN14 and *Lactobacillus rhamnosus* BGT10 in different experimental settings, which included the variation of strain ratio, total lactobacilli concentration, percentage of moisture and duration of incubation. Short peptides, soluble proteins, phospho-, neutral and unsaturated lipids were quantified. Differences among treated and control feeds were evaluated by Student t-test, while Gaussian process regression (GPR) modeling was employed to simulate the incubation process and define the optimal treatment combination in the context of overall feed nutritional profile. Treatment duration was shown to be the critical determinant of final outcome, either as single factor or via interaction with strain ratio. Optimal nutrient balance was achieved with 12 h incubation period, 260% moisture, 75:25 and 50:50 BGHN14:BGT10 ratios and 200 mg of lactobacilli per g of dry feed. This study should serve as the basis for large-scale tests which would simulate on-farm production of both fishmeal-based and unconventional, lower cost aquafeed with added value.

## Introduction

Intensive rearing of fish in closed system, where natural ecosystem is missing, is significant task in terms of provision of satisfactory balance of nutrients needed to sustain fish development and growth [1]. This is of special importance for carnivorous fish, which demand higher quality feed rich in lipids and proteins to satisfy energetic demands and maintain uninterrupted growth [2, 3]. Though very expensive, commercial fishmeal-based larval feeds still cannot completely support fish development, which predisposes fish to high mortalities after early weaning [4, 5, 6]. This is partly due to high resistance of commercial feed to physical breakdown and ineffectiveness of mechanical digestion processes in larval fish [7, 8]. Commercial feed also contains lower percentage of soluble proteins when compared to commonly used live foods, which further aggravates nutrient assimilation, given the low digestive protease activity in larval gut [9, 10, 11, 12]. Additionally, because of specific processes applied during feed manufacturing, like heating and fat coating, commercial feed is usually presented with low phospholipid and excess neutral lipid amount on its surface [13, 14, 15]. This impedes larval development via interference with free fatty acid (FFA) absorption and induction of structural damage in enterocytes [16, 17].

Procedures like addition of phospholipids to artificial feed and incorporation of hydrolyzed diets, which increases feed protein solubility and digestibility [18, 19], are associated with high costs, preventing their wide-spread application in feed industry [20]. Microorganisms may represent more economically and ecologically viable option for modification of nutritive properties of artificial fish diet. In this respect, application of lactic acid bacteria (LAB), particularly lactobacilli, in aqua-feeds poses minimal risk both for fish and staff working in fish rearing facilities, given the qualified presumption of safety (QPS) status of most lactobacilli species [21]. Lactobacilli have been attributed with numerous probiotic effects in aquaculture, especially immune stimulation potential. This is of particular importance for larval fish, which have immature immune system [22, 23]. Although treatment of fishmeal with microorganisms, including lactobacilli, was proven to increase feed protein solubility/digestibility [24, 25, 26], so far there are no available data concerning their ability to balance the amounts of peptides/proteins, phospho-, neutral and unsaturated lipids in artificial fish diet.

Having in mind above explained problems concerning nutritive demands of larval carnivorous fish, the aim of present study was to evaluate at a laboratory scale whether solid state treatment with lactobacilli may alter the composition of nutrients in fishmeal-based larval feed. The modifications were directed towards the surface segment of feed granules, which makes the most relevant portion of particulate feed for larval fish, because of low efficiency of mechanical feed digestion in larval alimentary tract. The combination of two lactobacilli strains: *Lactobacillus paracasei* subsp. *paracasei* BGHN14 and *Lactobacillus rhamnosus* BGT10, which already showed the ability to hydrolyze proteins in fishmeal-based diet [27], was used. Different parameters related to lactobacilli application were varied, including total bacterial concentration, BGHN14 to BGT10 ratio, percentage of added saline and duration of incubation. Our results suggest the ability of lactobacilli to improve nutrient balance in the surface portion of feed granules without disrupting their granular shape. Possible mechanisms underlying observed changes are discussed.

## Materials & methods

### Bacteria used in the study

Combination of two strains: *Lactobacillus paracasei* subsp. *paracasei* BGHN14 and *Lactobacillus rhamnosus* BGT10 was used in the experiment. Both bacterial strains belong to bacterial collection of Laboratory for Molecular Microbiology (LMM), IMGGE, University of Belgrade, Serbia. For feed treatment, bacteria were cultured until late logarithmic growth phase: 10 h for BGHN14 at 30°C and 12 h for BGT10 at 37°C in aerobic atmosphere. Cultivation of both strains was done in De Man, Rogosa and Sharpe medium (MRS) (Oxoid, Hamshire, UK) as explained previously [27].

### Feed treatment

Dry feed used in the research was OTOHIME B1 larval feed (size ~360 μm) (Marubeni Nishin Feed Co., Ltd., Tokyo, Japan) containing significant percentage of fishmeal. Treatment of OTOHIME B1 with lactobacilli was performed in empty Petri dish. Since any disruption of feed granule integrity leads to poor feed acceptance by fish, feed treatment was performed in a manner that maximally preserves their granular shape. Feed granules were evenly spread in thin layer on the surface of Petri dish, before the addition of lactobacilli suspensions. This way, contacts between feed granules were very low, preventing their extensive agglutination after wetting. Feed particles were homogenously mixed with lactobacilli suspensions using inoculation loop. Four factors were varied during treatment:

1. Duration of incubation with lactobacilli—12 h or 24 h.
2. Lactobacilli concentration (biomass per mass of dry OTOHIME B1) - 5 or 10 mg of wet culture pellets per 50 mg of dry feed (100 mg or 200 mg per 1 g). The number of BGHN14 and BGT10 cells per 1 mg of wet pellet was ~5×10^8^ and 3×10^8^, respectively.
3. Ratios of BGHN14 to BGT10 pellet mass mixed with feed granules: 75:25, 50:50 or 25:75.
4. Moisture level–saline or lactobacilli suspensions in saline were added to obtain final 260% v w^-1^ or 390% v w^-1^ (volume per weight of dry feed).

In order to minimize the variance in results due to technical errors, experiments were performed at a small scale and all treatments and subsequent manipulations for all combinations with same incubation period (12 or 24 h) were performed simultaneously. Treatment of feed granules was performed as follows: wet pellets of BGHN14 and BGT10 cultures were mixed at one of the three above ratios (75:25, 50:50 and 25:75) to give totally 5 or 10 mg total pellet mass per feed sample. This pellet mass was suspended in 130 or 195 μL of saline per sample and mixed with 50 mg od dry feed granules spread onto the surface of Petri dish, as explained above. Controls mixed with saline (130 or 195 μL) for each incubation periods were included. This gave 14 treatment combinations per one incubation period (12 or 24 h) with three replicates per group. After addition of lactobacilli (or saline), Petri dishes were closed and wrapped in foil (to reduce evaporation) and incubated at 37°C for 12 or 24 h. Afterwards, small amounts of wet feed were taken for microbiological assays and the rest was air-dried at 50°C for 4 h. All treatments were performed in triplicates.

Extraction of lipids from dried whole, non-homogenized granules was done by addition of 20 volumes of 2:1 chloroform: methanol to 10 mg of feed granules per sample (Folch extraction) [28]. After 1 h incubation at room temperature (RT) with shaking and centrifugation at 14 500 RCF (relative centrifugal force) for 10 min at RT, supernatants were divided equally in two tubes. Pellets and both fractions of supernatants were vacuum-desiccated. Pellets were used for peptide and protein measurements. Lipids remaining in desiccated supernatants were used for phospho, neutral and unsaturated lipid assays (methodologies provided below). As explained above, extractions and measurements for each analyzed parameter (below) for all samples with the same incubation period (12 or 24 h) were performed simultaneously. In parallel, dry control samples were subjected to extraction and measurements for each series of samples.

### Free amino acid (FAA)/short peptide and soluble protein measurement

Defatted feed granules were resuspended in 20 volumes of distilled water and incubated for 10 min with shaking at RT, to extract water-soluble material from surface of granules. After centrifugation for 1 min at 14 500 RCF, RT, supernatants were analyzed for soluble protein content using Bradford assay with commercial Bradford reagent. In parallel, defined volume of supernatant was used for FAA/SP measurement by trinitrobenzene sulfonate (TNBS) assay [29]: supernatants were precipitated with 5 volumes of trichloroacetic acid (TCA) on ice for 15 min for FAA/short peptide assay, centrifuged for 10 min at 14 500 RCF, and mixed with 10 volumes of 0.1 M sodium phosphate buffer pH 8.0 plus 5 volumes of 0.1% TNBS dissolved in the same buffer. The mixture was incubated at 60°C in the dark for 45 min. Afterwards, reaction was stopped by addition of 0.25 M HCl and the absorbance at 420 nm was measured. Standard curves were made using DL-alanine and bovine serum albumin (BSA) for TNBS and Bradford assay, respectively.

### Phospholipid measurement

Lipid pellets obtained from one fraction of supernatants after lipid extraction were resuspended in chloroform for analysis of phospholipid amount by ammonium thiocyanate assay [28]. Ammonium thiocyanate reagent was prepared by dissolving 0.27 g of FeCl_3_ x 6H_2_O with 0.3 g of NH_4_SCN in 10 mL of distilled water. The reagent was mixed with lipid samples in 1:2 ratio, shaken and centrifuged at low speed for 1 min to separate lipid and water phases. Lower phase was transferred to new tubes and vacuum-desiccated. Pellets were resuspended in 96% of ethanol and solubilized by warming at 42°C. Absorbance at 488 nm was measured. L-phosphatidylcholine from egg yolk was used for standard curve preparation.

### Neutral lipid measurement

Lipid pellets obtained from the second fraction of supernatant were resuspended in 20% ethanol, warmed at 42°C until full solubillization and then used for neutral and unsaturated lipid measurements. Nile Red was used for neutral lipid analysis [30]. Samples were 20-fold diluted and transferred to dark microplates (200 μL) for fluorescence measurement. Then, 3 μL of 0.25 mg mL^-1^ Nile Red/acetone solution was added; measurement started immediately at 37°C and was continued for the next 20 min every 1 min (480 nm excitation wavelength and 570–580 nm emission wavelength). Differences between the last and first measurement point were calculated. Standard curve was prepared using glyceryl trioleate.

### Unsaturated lipid measurement

Colorimetric sulfo-phospho-vanillin (SPV) assay was used for unsaturated lipid analysis [31]. Samples prepared as described above were mixed at 1:10 ratio with sulfuric acid in glass tubes. After 10 min of boiling and cooling at RT, 2.5 volumes of phospho-vanillin (PV) reagent were added. PV reagent was prepared by mixing vanillin reagent (60 mg of vanillin per 1 L of water) and 85% phosphoric acid in 1:4 ratio. Mixture was incubated for 15 min at 37°C and then for 45 min in dark, RT. Absorbance was measured at 530 nm. Coconut oil was used for standard curve preparation.

### Microbiological analysis

Sterile saline was added to weighed wet feed in a ratio of 50:1 to finally reach 2% w v^-1^. Further dilutions in saline were made and 10 μL of prepared suspensions were inoculated onto the surface of universal Luria Agar (LA) medium [32]. Colonies were counted after 48 h of incubation at 37°C aerobically.

### Statistical analysis

For statistical analyses, samples with the same duration of incubation were calibrated against the mean value of three replicates obtained in simultaneously extracted and measured dry controls. Comparisons between treatments and dry or respective (260 or 390%) wet controls were performed by t-test. Normality of data in control and treatment groups was assessed by Shapiro-Wilk test. Since process modeling could not be performed by least square method, due to significant lack of fit, modeling was performed using Gaussian process regression (GPR) [33, 34], which does not have assumptions concerning the regression function distribution. Bayesian information criterion (BIC) and Pearson correlation coefficient between predicted and actual amounts for each analyzed parameter were calculated in order to evaluate model validity. Main and interaction effect sensitivity values were reported. Desirability analysis was performed in order to facilitate the selection of the level combination of factors that yields optimal nutritive profile of treated feed. GPR modeling, sensitivity analysis and graph drawing were performed using JMP11 (SAS Institute, Cary, NC, US). Student test, normality and Pearson correlation tests were done using SPSS 20 (IBM Corporation, Armonk, NY, US).

## Results and discussion

**Fig 1:**
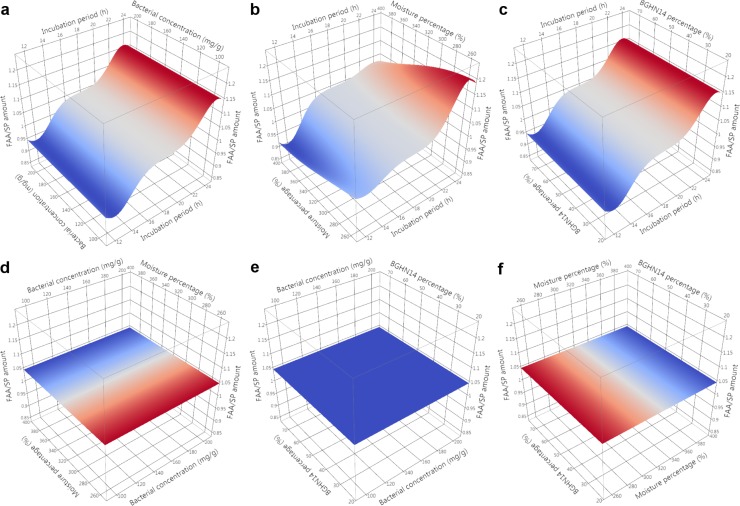
**GPR surface profilers showing simulated changes of intensities of free amino acids / short peptides (FAA/SP) as a function of: (a) incubation period (h) x bacterial concentration (mg g^-1^), (b) incubation period (h) x moisture percentage (%), (c) incubation period x BGHN14 percentage (%), (d) bacterial concentration (mg g^-1^) x moisture percentage (%), (e) bacterial concentration (mg g^-1^) x BGHN14 percentage (%) and (f) moisture percentage (%) x BGN14 percentage (%) existence of trend towards change of intensity at a given factor combinations is marked by different colors (blue minimum intensity, red maximum intensity), regardless of the intensity of response variable; GPR—Gaussian process regression**.

**Fig 2:**
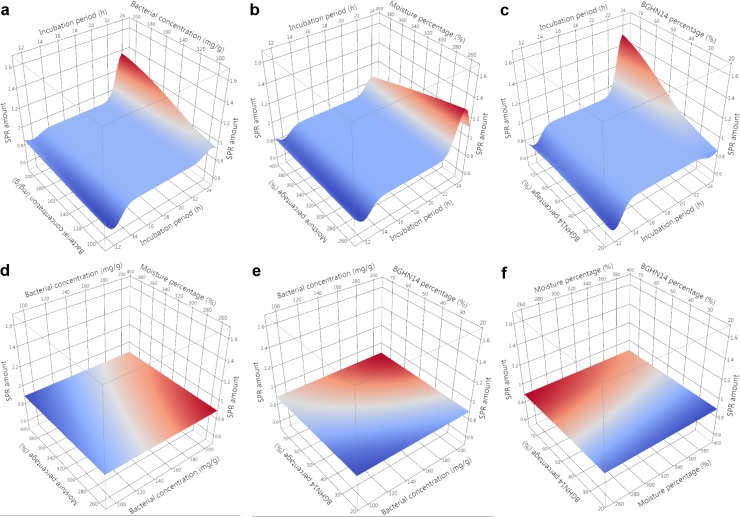
**GPR surface profilers showing simulated changes of intensities of soluble proteins (SPR) as a function of: (a) incubation period (h) x bacterial concentration (mg g^-1^), (b) incubation period (h) x moisture percentage (%), (c) incubation period x BGHN14 percentage (%), (d) bacterial concentration (mg g^-1^) x moisture percentage (%), (e) bacterial concentration (mg g^-1^) x BGHN14 percentage (%) and (f) moisture percentage (%) x BGN14 percentage (%); existence of trend towards change of intensity at a given factor combinations is marked by different colors (blue minimum intensity, red maximum intensity), regardless of the intensity of response variable; GPR—Gaussian process regression**.

**Fig 3:**
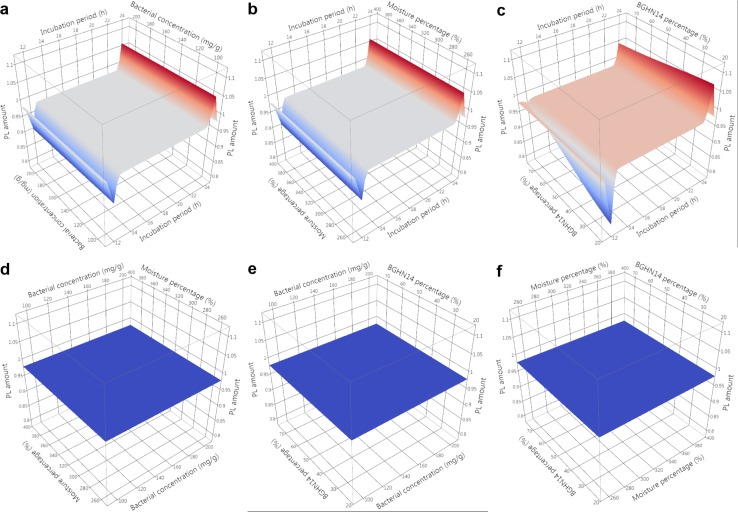
**GPR surface profilers showing simulated changes of intensities of phospholipids (PL) as a function of: (a) incubation period (h) x bacterial concentration (mg g^-1^), (b) incubation period (h) x moisture percentage (%), (c) incubation period x BGHN14 percentage (%), (d) bacterial concentration (mg g^-1^) x moisture percentage (%), (e) bacterial concentration (mg g^-1^) x BGHN14 percentage (%) and (f) moisture percentage (%) x BGN14 percentage (%); existence of trend towards change of intensity at a given factor combinations is marked by different colors (blue minimum intensity, red maximum intensity), regardless of the intensity of response variable; GPR—Gaussian process regression**.

**Fig 4:**
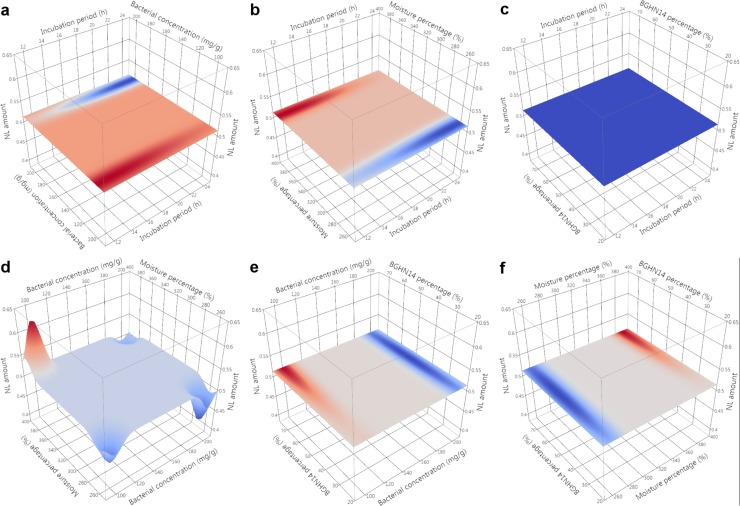
**GPR surface profilers showing simulated changes of intensities of neutral lipids (NL) as a function of: (a) incubation period (h) x bacterial concentration (mg g^-1^), (b) incubation period (h) x moisture percentage (%), (c) incubation period x BGHN14 percentage (%), (d) bacterial concentration (mg g^-1^) x moisture percentage (%), (e) bacterial concentration (mg g^-1^) x BGHN14 percentage (%) and (f) moisture percentage (%) x BGN14 percentage (%); existence of trend towards change of intensity at a given factor combinations is marked by different colors (blue minimum intensity, red maximum intensity), regardless of the intensity of response variable; GPR—Gaussian process regression**.

**Fig 5:**
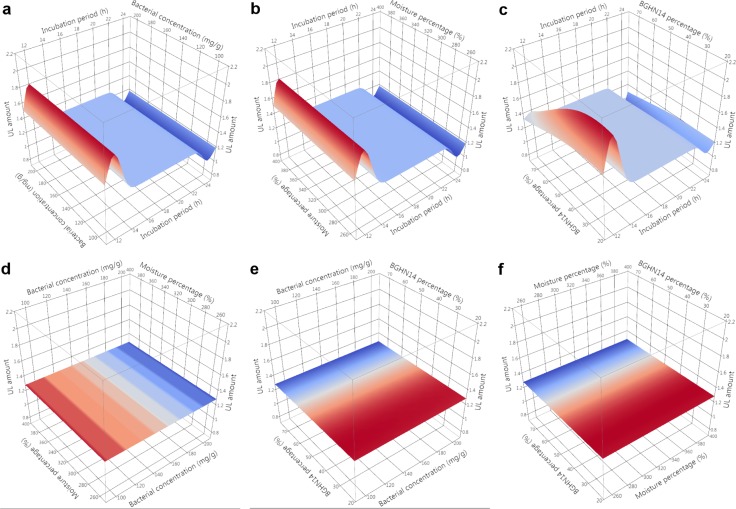
**GPR surface profilers showing simulated changes of intensities of unsaturated lipids (UL) as a function of: (a) incubation period (h) x bacterial concentration (mg g^-1^), (b) incubation period (h) x moisture percentage (%), (c) incubation period x BGHN14 percentage (%), (d) bacterial concentration (mg g^-1^) x moisture percentage (%), (e) bacterial concentration (mg g^-1^) x BGHN14 percentage (%) and (f) moisture percentage (%) x BGN14 percentage (%); existence of trend towards change of intensity at a given factor combinations is marked by different colors (blue minimum intensity, red maximum intensity), regardless of the intensity of response variable; GPR—Gaussian process regression**.

Evaluation of nutritive profile of fishmeal-based OTOHIME B1 feed granules after treatment with different BGHN14/BGT10 combinations was performed by measurement of levels of free amino acids (FAA)/short peptides (SP), soluble proteins (SPR), phospholipids (PL), neutral lipids (NL) and unsaturated lipids (UL). Absolute values of measurements are given in S1 Table. In order to compensate for differences arising from possible day-to-day variance in measurements, statistical comparisons and modeling were performed with results calibrated against the mean value of three replicates obtained with simultaneously extracted and measured dry controls. Mean ± standard error (SE) values of calibrated results with t-test results are presented in Tables 1 and 2. Normality test results are shown in S2 Table. Results of Gaussian process regression (GPR) sensitivity analysis along with model parameters are given in Table 3. GPR surface profilers are presented Figs 1–5 while GPR predictive profilers with optimal factor level combinations and respective desirability values are provided as S1 Fig and S2 Fig.

**Table 1:** Feed biochemical profile (calibrated values) after 12 h incubation period.

	Mean ± standard error of mean (SEM) (n = 3)				
	FAA/SP amount	SPR amount	PL amount	NL amount	UL amount
75:25, 260%, 100 mg g^-1^	1.09 ± 0.06	0.74 ± 0.07	1 ± 0.1	0.51 ± 0.06 * p = 0.038	1.27 ± 0.26
50:50, 260%, 100 mg g^-1^	0.74 ± 0.15	0.78 ± 0.06	0.87 ± 0.04	0.47 ± 0.09 * p = 0.038	1.7 ± 0.14
25:75, 260%, 100 mg g^-1^	0.82 ± 0.01	0.54 ± 0.12 * p = 0.033	0.77 ± 0.06	0.58 ± 0.03 * p = 0.049	2.53 ± 0.61
75:25, 260%, 200 mg g^-1^	1.11 ± 0.14	0.49 ± 0.1 * p = 0.014	0.95 ± 0.06	0.36 ± 0.06 * p = 0.016, ^#^ p = 0.017	0.71 ± 0.08
50:50, 260%, 200 mg g^-1^	0.95 ± 0.06	1.06 ± 0.18	0.85 ± 0.04	0.39 ± 0.04 * p = 0.017, ^#^ p = 0.015	1.96 ± 0.61
25:75, 260%, 200 mg g^-1^	1.2 ± 0.11	0.5 ± 0.04 * p = 0.004	0.79 ± 0.08	0.52 ± 0.16	1.56 ± 0.16
260% control	0.9 ± 0.07	0.96 ± 0.16	0.89 ± 0.01	0.66 ± 0.05	3.69 ± 1.63
75:25, 390%, 100 mg g^-1^	0.86 ± 0.07	0.81 ± 0.06	1.03 ± 0.16	0.6 ± 0.11	1.68 ± 0.38
50:50, 390%, 100 mg g^-1^	1.08 ± 0.2	0.57 ± 0.18	1.02 ± 0.13	0.6 ± 0.16	2.15 ± 0.66
25:75, 390%, 100 mg g^-1^	0.77 ± 0.07	0.64 ± 0.16	0.81 ± 0.03	0.63 ± 0.03	1.94 ± 0.84
75:25, 390%, 200 mg g^-1^	0.83 ± 0.05	0.72 ± 0.05 * p = 0.037	0.86 ± 0.01	0.66 ± 0.13	1.62 ± 0.32
50:50, 390%, 200 mg g^-1^	0.87 ± 0.03	0.87 ± 0.17	0.96 ± 0.07	0.53 ± 0.06 * p = 0.042	1.8 ± 0.54
25:75, 390%, 200 mg g^-1^	0.83 ± 0.05	0.88 ± 0.17	0.79 ± 0.1	0.53 ± 0.05 * p = 0.04	2.39 ± 0.84
390% control	0.81 ± 0.2	0.78 ± 0.1	0.8 ± 0.07	0.69 ± 0.07	2.94 ± 0.79
Dry control	1 ± 0.09	1 ± 0.07	1 ± 0.08	1 ± 0.15	1 ± 0.34

Mean values of calibrated FAA/SP, SPR, PL, NL and UL amounts in 12 h lactobacilli treated and control feed

* and # indicate statistically significant (p < 0.05) differences relative to dry and relevant (260 or 390%) wet control, respectively (comparisons were made only between treatments and control groups); means with statistically significant differences relative to dry control are bolded; FAA/SP—free amino acids/short peptides; SPR—soluble proteins; PL—phospholipids; NL—neutral lipids; UL—unsaturated lipids; n—replicates per treatment group.

**Table 2:** Feed biochemical profile (calibrated values) after 24 h incubation period.

	Mean ± standard error of mean (SEM) (n = 3)				
	FAA/SP amount	SPR amount	PL amount	NL amount	UL amount
75:25, 260%, 100 mg g^-1^	1.33 ± 0.15	1.21 ± 0.07	1.11 ± 0.09	0.39 ± 0.1 * p = 0.007	0.71 ± 0.41
50:50, 260%, 100 mg g^-1^	1.18 ± 0.07	1.1 ± 0.08	1.03 ± 0.05	0.33 ± 0.02 * p = 0.001, ^#^ p = 0.032	1.56 ± 0.54
25:75, 260%, 100 mg g^-1^	1.15 ± 0.2	0.82 ± 0.08	1.06 ± 0.03	0.43 ± 0.09 * p = 0.006	1.18 ± 0.58
75:25, 260%, 200 mg g^-1^	1.27 ± 0.05 * p = 0.014	2.18 ± 0.39 ^#^ p = 0.024	1.07 ± 0.03	0.55 ± 0.09 * p = 0.015	0.58 ± 0.24
50:50, 260%, 200 mg g^-1^	1.13 ± 0.11	1.38 ± 0.06 ^#^ p = 0.012	1.02 ± 0.04	0.33 ± 0.07 * p = 0.002, ^#^ p = 0.046	0.54 ± 0.25
25:75, 260%, 200 mg g^-1^	1.37 ± 0.23	1.14 ± 0.05 ^#^ p = 0.044	1.1 ± 0.04	0.42 ± 0.11 * p = 0.011	1.17 ± 0.03
260% control	0.99 ± 0.07	0.72 ± 0.14	1.15 ± 0.04	0.72 ± 0.12	1.51 ± 0.31
75:25, 390%, 100 mg g^-1^	1.04 ± 0.12	1.06 ± 0.03 ^#^ p = 0.007	1.09 ± 0.03	1.05 ± 0.15	0.87 ± 0.1
50:50, 390%, 100 mg g^-1^	1.24 ± 0.13 ^#^ p = 0.02	0.99 ± 0.06 ^#^ p = 0.013	1.05 ± 0.02	0.53 ± 0.04 * p = 0.004, ^#^ p = 0.015	1.61 ± 0.63
25:75, 390%, 100 mg g^-1^	1.04 ± 0.07 ^#^ 0.019	0.71 ± 0.15	1.1 ± 0.03 * p = 0.019	0.53 ± 0.13 * p = 0.03	0.89 ± 0.16
75:25, 390%, 200 mg g^-1^	1.08 ± 0.04 ^#^ p = 0.005	1.48 ± 0.26 ^#^ p = 0.021	0.96 ± 0.03	0.3 ± 0.14 * p = 0.011, ^#^ p = 0.027	0.38 ± 0.23
50:50, 390%, 200 mg g^-1^	1.19 ± 0.09 ^#^ p = 0.008	1.15 ± 0.11 ^#^ p = 0.011	1.03 ± 0.02	0.36 ± 0.06 * p = 0.002, ^#^ p = 0.005	1.3 ± 0.86
25:75, 390%, 200 mg g^-1^	0.88 ± 0.05	0.78 ± 0.17	1.13 ± 0.04 * p = 0.043	0.42 ± 0.1 * p = 0.008, ^#^ p = 0.025	0.7 ± 0.09
390% control	0.7 ± 0.05 * p = 0.009	0.39 ± 0.13 * p = 0.049	1.24 ± 0.22	0.81 ± 0.06	3.34 ± 1.82
Dry control	1 ± 0.04	1 ± 0.18	1 ± 0	1 ± 0.07	1 ± 0.08

Mean values of calibrated FAA/SP, SPR, PL, NL and UL amounts in 24 h lactobacilli treated and control feed

* and # indicate statistically significant (p < 0.05) differences relative to dry and relevant (260 or 390%) wet control, respectively (comparisons were made only between treatments and control groups); means with statistically significant differences relative to dry control are bolded; FAA/SP—free amino acids/short peptides; SPR—soluble proteins; PL—phospholipids; NL—neutral lipids; UL—unsaturated lipids; n—replicates per treatment group.

**Table 3:** Sensitivity analysis results.

		Total Sensitivity	Main Effect	Incubation period Interaction	Bacterial concentration Interaction	Moisture percentage Interaction	BGHN14:BGT10 ratio Interaction
**FAA/SP** BIC = 1.49 Pearson's r = 0.552 (p < 0.001)	Incubation period	0.96	0.923	0	0	0.0364	0
	Bacterial concentration	0	0	0	0	0	0
	Moisture percentage	0.077	0.04	0.0364	0	0	0
	BGHN14:BGT10 ratio	0	0	0	0	0	0
**SPR** BIC = 54.62 Pearson's r = 0.827 (p < 0.001)	Incubation period	0.944	0.682	0	0.056	0.024	0.182
	Bacterial concentration	0.07	0.013	0.056	0	0	0
	Moisture percentage	0.026	0.002	0.024	0	0	0
	BGHN14:BGT10 ratio	0.213	0.031	0.182	0	0	0
**PL** BIC = -83.77 Pearson's r = 0.684 (p < 0.001)	Incubation period	0.997	0.876	0	0	0	0.121
	Bacterial concentration	0	0	0	0	0	0
	Moisture percentage	0	0	0	0	0	0
	BGHN14:BGT10 ratio	0.124	0.003	0.121	0	0	0
**NL** BIC = -18.83 Pearson's r = 0.694 (p < 0.001)	Incubation period	0.019	0.002	0	0.009	0.007	0
	Bacterial concentration	0.689	0.063	0.009	0	0.612	0.004
	Moisture percentage	0.713	0.087	0.007	0.612	0	0.007
	BGHN14:BGT10 ratio	0.012	0.001	0	0.004	0.007	0
**UL** BIC = 192.96 Pearson's r = 0.547 (p < 0.001)	Incubation period	0.976	0.878	0	0	0	0.098
	Bacterial concentration	0	0	0	0	0	0
	Moisture percentage	0	0	0	0	0	0
	BGHN14:BGT10 ratio	0.122	0.024	0.098	0	0	0

Sensitivity indices for process factors calculated from Gaussian process regression (GPR) model simulating the incubation of feed with lactobacilli; model parameters, including Bayesian information criterion (BIC) and predicted-observed values correlations for each analyzed variable are provided below variable designations; FAA/SP—free amino acids/short peptides; SPR—soluble proteins; PL—phospholipids; NL—neutral lipids; UL—unsaturated lipids.

Nutritive demands of carnivorous larval fish differ as compared to adult fish, especially because stomach, which represents the main place of protein digestion, in most fish species does not develop until juvenile stage, predisposing the larvae to poor protein digestion capacity [35, 36]. Inclusion of protease digested meal, which is expected to contain high levels of soluble proteins, was evaluated earlier for improvement of fish growth after transition to artificial diet [37]. Though promising, these results suggest that limited proteolysis is superior over extensive proteolysis when it comes to fish growth, presumably due to high nutrient leaching to the water after excessive hydrolysis [38, 39]. The degree of nutrient loss from feed granules is dependent on molecule size and solubility [10]. In present study, levels of both FAA/SP and SPR in lactobacilli treated feed were evaluated, to discriminate between moderate and excess proteolysis. Though both FAA/SP and SPR are considered to be beneficial for enhancement of feed nutritive value, FAA/SP will probably leach into the circulating water due to small size; this would not only reduce the amount of nutrients available to fish, but might also lead to overgrowth of bacteria in tank water. Therefore, too big FAA/SP increase was considered as unfavorable treatment outcome in present research. According to the results of t-test and GPR, 24 h incubation with lactobacilli was shown to increase SPR level, especially with 75:25 combinations, indicating involvement of BGHN14 in protein solubilization. Lactobacilli were previously reported to increase the solubility of proteins in feed either via lactic acid production which changes protein-water interaction or via direct proteolysis of water-insoluble proteins [40, 41]. Although 75:25 24 h combinations were shown to improve feed SPR composition, this was paralleled by simultaneous increase of FAA/SP amount, presumably as the result of hydrolysis of SPR by added lactobacilli or feed-derived proteases. In fact, the hydrolysis of SPR occurred much faster than protein solubilization, as evident from the results of 12 h incubation of feed with lactobacilli, showing significant drop of SPR amount in comparison with controls, especially with 200 mg g^-1^ combinations. The observed SPR drop in 12 h lactobacilli-treated feed samples may be the result of activity of lactobacilli-derived proteases or the stimulation of feed-derived proteases by lactobacilli activity, as already reported by Yin et al. [42]. Endogenous feed proteases might have also been activated in the face of increased moisture percentage [43], which is evident from the results of SPR analysis in 24 h, 390% moisture control feed samples. Additionally, leaching of SPR to the surface of Petri dish during feed treatment due to high moisture percentage should not be excluded. In support of this, FAA/SP were also decreased in the same treatment group as compared to dry control feed.

Aside from proteins, lipids play an important role in development of fish, especially nervous and skeletal system. The availability of free fatty acids (FFA) is dependent on the class of lipids acting as FFA carriers. FFA present in PL are better absorbed in fish gut, because PL play crucial role in transport of lipids from enterocytes to circulation [44]. Since this process is not fully functional in larval fish, which have limited capacity to *de novo* synthesize PL [17, 45], their PL demands may go up to 12% [46]. Although fishmeal contains approximately 5% PL [46], further increase of PL in artificial diet would beneficially affect FFA assimilation by fish larvae. GPR revealed slight sensitivity of PL content to strain ratio in 12 h lactobacilli treated samples, with 25:75 ratio correlating with lower PL content. Alongside with this, there were tendencies towards drop of PL content in 260 and 390% moisture, 100 mg g^-1^ concentration, 25:75 strain ratio combination relative to dry control (p = 0.077 and 0.079, respectively). This drop was also noticed with other 12 h treated 25:75 strain ratio combinations but no significance or tendency was reached. Since same, though insignificant, drop of PL content was observed in 12 h incubated wet controls, PL degradation is probably the result of activation of endogenous feed-derived phospholipases, which was enhanced in the presence of BGT10. Phenotypic appearance of BGT10 in liquid culture and agar plate indicated that BGT10 secretes exopolysaccharide (EPS), a high molecular weight biopolymer reported to be produced by many lactic acid bacterial strains [47]. Though additional confirmation at chemical level is needed, bacterial EPS may act as anionic emulsifier [48] and might have facilitated phospholipase activity against feed derived PL [49]. According to GPR, PL level was highly sensitive to incubation period, with 24 h incubation of feed with lactobacilli yielding higher PL level than 12 h incubation. This was supported by significant increase of PL, especially with 390% combinations in comparison with dry controls. Since the tendency towards PL increase was also seen in 260% wet control samples (p = 0.064), it is possible that water addition enhanced lipid extraction, as reported previously by Ren and co-workers [50]. Hypothetically, this may reflect the bioavailability of PL for larval fish, with increased extractability leading to enhanced feed value. Contrary to 12 h incubation period, GPR modeled PL level in 24 h incubation regimen was not sensitive to change of strain ratio.

Although NL represent energy dense substrates and may provide benefits for carnivorous fish, high level of NL in larval fish diet may negatively affect FFA absorption [17]. Artificial feed pelleting/extrusion commonly involves the addition of lipid coat for improvement of its water stability and floating characteristics [15]. Hence, the amount of neutral lipids on the surface of feed granules/pellets which initially comes into contact with digestive enzymes in fish gut may be critically high. Reduction of NL in artificial feed would beneficially affect feed assimilation after weaning. Tendency towards NL decrease was seen in 260% wet control feed samples (p = 0.097), probably as the result of activation of feed-derived lipases. Addition of lactobacilli correlated with further decrease of NL level, compared both to dry and wet controls samples. GPR modeling showed an interaction between the moisture percentage and lactobacilli concentration in determination of NL content in lactobacilli treated feed, with 100 mg g^-1^, 390% moisture combinations correlating with lowest NL drop. Lipase activation was shown to be dependent on hydration level; however, this activity is not linear, but rather dependent on the size of lipid-water interface: in solutions with high water content, lipid-water interface would be small with lipases predominantly present in the water phase and inactive [51]. Therefore, aside from possible lactobacilli-derived lipase activity which might have contributed to NL degradation [49, 52], lactobacilli cells might have acted as water absorbing units. This may explain why a decrease of NL quantity was least pronounced with 390% moisture, 100 mg g^-1^ combinations. In line with this, 24 h incubated 390% moisture, 100 mg g^-1^ concentration, 75:25 strain ratio combination was not presented with lower NL level as compared to dry control, though there was a tendency towards NL decrease in the same combination after 12 h incubation period (p = 0.092). Lactobacilli have not been ascribed the ability to deposit neutral lipids [53], so it is unlikely that observed elevation of triglycerides is the result of deposition caused by added bacteria. Eventually, degradation of proteins present in lipoprotein complexes might have released lipids and facilitate lipid extraction [54]. As reported above, 24 h treated 75:25 strain ratio combinations were presented with significant protein solubilization/degradation, which probably increased NL extractability and, in concert with low NL degradation rate seen with 390% moisture, 100 mg g^-1^ concentration combinations, resulted in the raise of NL amount with prolongation of incubation in 75:25 ratio, 390% moisture, 100 mg g^-1^ concentration combination treated feed. This would probably affect the amount of NL available to larval fish after feed ingestion.

Degradation of NL may lead to extensive loss of unsaturated free fatty acids (FFA), due to their liberation from lipid complexes and consequential oxidation [55]. Prevention of oxidation and loss of unsaturated fats along with fat hydrolysis, as observed here for NL, represents an important step towards production of high-quality fish diet [56]. Presence of unsaturated FFA in diet is of utmost importance for carnivorous fish, given their lower capacity to synthesize unsaturated fats as compared to herbivorous fish [57]. Deficiency of unsaturated fats in fish diet, in concert with reduced PL content, was linked to development of numerous skeletal anomalies, leading to high fish mortality [58]. Statistical analysis revealed a tendency towards an increase of UL percentage in 390% controls (p = 0.088), as compared to dry controls. UL increase may be the result of activation of feed-derived desaturases after water addition, as was also observed during frozen meat storage [59]. However, an increase of UL after water addition seemed to be counteracted by lactobacilli addition, though, due to large deviations, this was supported only by statistical tendencies with 24 h treated samples. In 12 h incubation regimen, UL level was slightly affected by strain ratio, with 75:25 combinations leading to highest interference with UL increase, indicating that BGHN14 may be responsible for observed effects. Consumption of oxygen by lactobacilli or eventual sequestration of cofactors needed for desaturase activity, are some of the reasons that might have contributed to the reduction of enzyme activity after lactobacilli addition [60, 61]. Furthermore, some lactobacilli strains were shown to increase the oxidative deterioration of lipids in meat products, via hydrogen peroxide production [62]. According to GPR sensitivity analysis, 24 h incubation period with lactobacilli correlated with further reduction of UL amount, presumably as the result of continued FFA oxidation with prolonged incubation.

Though feed drying after treatment significantly reduces the viability of microorganisms, microbiota content in treated feed prior to feed drying was analyzed in order to check for possible presence of pathogen-derived toxins/metabolites in treated diet. Since no precise quantitative comparison of microbial count among treatments can be obtained with wet samples, this was taken as rough estimation of microbial growth. Analysis of microbiota content in wet feed on universal LA medium revealed no significant growth of bacteria (other than lactobacilli) in 12 h incubation regime, while in some samples of 24 h incubated feed, growth of non-lactobacilli and/or yeasts was detected. Eventual usage of extruded diet may lower the risk of microbial growth. Furthermore, activation of internal feed-derived and contaminating microbe enzymes would be lowered in extruded diet, thus allowing for tighter control of the entire treatment process.

Final selection of optimal treatment regimen, which would enhance overall nutrient bioavailability, was based on evaluation of balance among analyzed parameters. According to above presented results, 24 h incubation period and BGHN14 dominance were shown to positively affect SPR amount, though this was paralleled by an increase of FAA/SP. Similarly, an increase of incubation duration correlated with elevation of PL content, while BGHN14 prevalence prevented PL autolysis. However, shortening of incubation seemed to affect positively UL amount, which, on the other side, was negatively affected by BGHN14 presence. NL level was shown to be highly reduced with 260% moisture, 200 mg g^-1^ concentrations. Considering the results of microbial analysis and above explanations, it comes out that 12 h, 260% moisture, 200 mg g^-1^ concentration, 75:25 and 50:50 strain ratio combinations have the best capacity to regulate feed lipid and protein content. To assist the selection of optimal level combination(s), desirability levels of GPR were calculated. The peak of desirability (0.569) was obtained with 24 h, 260% moisture, 200 mg g^-1^ concentration, 56:44 strain ratio combination, with 50:50 and 75:25 strain ratio combinations giving 0.567 and 0.564 desirability levels, respectively. Desirability analysis with fixed 12 h incubation period yielded 260% moisture, 200 mg g^-1^ concentration, 56:44 strain ratio as the best treatment combination (desirability level 0.499), with 50:50 and 75:25 combinations giving 0.498 and 0.485 desirability levels, respectively. Due to low maximal desirability levels (below 0.5) obtained with these combinations, further modifications of experimental procedures are needed, such as coating of feed granules with materials which would minimize the leaching of water soluble materials or inclusion of lactobacilli strains with antioxidant and/or antibacterial properties which would potentially increase overall UL level and prevent the microbial growth, respectively. Technological improvements related to the manner of lactobacilli application to feed granules without their extensive agglutination should also be addressed in future studies. Although additional optimization is necessary, this small-scale research has demonstrated that lactobacilli may increase the bioavailability of nutrients present in the surface portion of fishmeal-based feed granules, while preserving granule structural integrity. Furthermore, given the ability of both viable and non-viable lactobacilli to stimulate fish immunity and infection resistance [63], lactobacilli enriched feed granules could have added probiotic value for larval fish. This opens the way for eventual application of lactobacilli for improvement of nutrient availability in lower quality, animal by-product based, larval feed [64, 65, 66], which would minimize the costs associated with larval rearing without profit reduction.

## Supporting information

S1 Fig Predictive profilers showing GPR modeled data with combinations of factor levels giving maximal overall desirability; graphs are vertically arranged according to analyzed variables (from top to bottom: FAA/SP, SPR, PL, NL, UL and values of desirability for each process factor) and horizontally according to process factors (from left to right: incubation period (h), bacterial concentration (mg g-1), moisture percentage (%), BGHN14 percentage (%) and values of desirability for each analyzed variable); desirability values were set to increase with increasing SPR, PL and UL amount, to decrease with increasing NL amount, while for FAA/SP desirability profile was set to assume hyperbolic shape reaching the peak at FAA/SP amount 1 and mild decrease with further elevation of FAA/SP amount; FAA/SP—free amino acids/short peptides; SPR—soluble proteins; PL—phospholipids; NL—neutral lipids; UL—unsaturated lipids; GPR—Gaussian process regression. (PDF)

S2 Fig Predictive profilers showing GPR modeled data with combinations of factor levels giving maximal desirability when incubation period was fixed to 12 h; graphs are vertically arranged according to analyzed variables (from top to bottom: FAA/SP, SPR, PL, NL, UL and values of desirability for each process factor) and horizontally according to process factors (from left to right: incubation period (h), bacterial concentration (mg g-1), moisture percentage (%), BGHN14 percentage (%) and values of desirability for each analyzed variable); desirability values were set to increase with increasing SPR, PL and UL amount, to decrease with increasing NL amount, while for FAA/SP desirability profile was set to assume hyperbolic shape reaching the peak at FAA/SP amount 1 and mild decrease with further elevation of FAA/SP amount; FAA/SP—free amino acids/short peptides; SPR—soluble proteins; PL—phospholipids; NL—neutral lipids; UL—unsaturated lipids; GPR—Gaussian process regression. (PDF)

S1 Table Feed biochemical profile (absolute values of measurements). Mean values of absolute FAA/SP, SPR, PL, NL and UL amounts in 12 h (a) and 24 h (b) lactobacilli treated and control feed; values are given as μg per mg of dried feed; FAA/SP—free amino acids/short peptides; SPR—soluble proteins; PL—phospholipids; NL—neutral lipids; UL—unsaturated lipids.
(PDF)

S2 Table Normality test results. Significance values (p) of Shapiro-Wilk test assessment of data normality in different treatment groups; p values higher than 0.05 indicate normal data distribution; FAA/SP—free amino acids/short peptides; SPR—soluble proteins; PL—phospholipids; NL—neutral lipids; UL—unsaturated lipids.
(PDF)

## References

[1] Craig S, Helfrich LA. Understanding fish nutrition, feeds, and feeding. Virginia Cooperative Extension (VCE), 2002; 420–256: 1–4.

[2] Andersen SM, Waagbø R, Espe M. Functional amino acids in fish nutrition, health and welfare. Front Biosci (Elite Ed). 2016; 8: 143–169. 26709652 10.2741/757

[3] Refstie S, Storebakken T. Vegetable protein sources for carnivorous fish: potential and challenges. Recent Advances in Animal Nutrition in Australia. 2001; 13: 195–203.

[4] Kolkovski S, Curnow J, King J. Development towards commercialization of marine fish larvae feeds—Microdiets. Project No. 2004/258. Department of Fisheries, Western Australia, Fisheries Research Report. 2010; 198.

[5] New MB, Csavas I. 1993. Aquafeeds in Asia—a regional overview. In: New MB, Tacon AGJ, Csavas I, editors. Farm-made Aquafeeds, Proceedings of the FAO/AADCP Regional Expert Consultation on Farm-Made Aquafeeds. FAO Fisheries Technical Paper. 1994; 343: 1–23. ISBN 92-5-103597-0

[6] Holt GJ, Webb KA, Rust MB. Microparticulate Diets: Testing and Evaluating Success In: Larval Fish Nutrition. Holt GJ, editor. New Jersey, US: John Wiley & Sons, Inc 2011; pp: 353–372. 10.1002/97804709598627

[7] Kolkovski S. Microdiets as alternatives to live feeds for fish larvae in aquaculture: Improving the efficiency of feed particle utilization In: Allan G, Burnell G, editors. Advances in Aquaculture Hatchery Technology. Sawston, UK: Woodhead Publishing Limited 2013; pp. 203–222. 10.1533/9780857097460.1.203

[8] Langdon C, Barrows R. Microparticulate diets: technology In: Holt GJ, editor. Larval Fish Nutrition. Hoboken, US: Wiley-Blackwell 2011; p. 335. ISBN 978-0-8138-1792-7

[9] Zambonino Infante JL, Gisbert E, Sarasquete C, Navarro I, Gutiérrez J, Cahu CL. Ontogeny and physiology of the digestive system of marine fish larvae In: Cyrino JEP, Bureau DP, Kapoor BG, editors. Feeding and Digestive Functions of Fishes. Boca Raton, US: CRC Press 2008; pp. 281–348. ISBN 9781578083756

[10] Hamre K, Yúfera M, Rønnestad I, Boglione C, Conceição LEC, Izquierdo M. Fish larval nutrition and feed formulation: knowledge gaps and bottlenecks for advances in larval rearing. Rev Aquacult. 2013; 5: S26–S58. 10.1111/j.1753-5131.2012.01086.x

[11] Samuelsen TA, Oterhals Å. Water‐soluble protein level in fishmeal affects extrusion behaviour, phase transitions and physical quality of feed. Aquac Nutr. 2016; 22: 120–133. 10.1111/anu.12235

[12] Vikas PA, Chakraborty K, Sajeshkumar NK, Thomas PC, Sanil NK, Vijayan KK. Quality of six indian populations of *Artemia franciscana* for larval finfish culture. J Appl Aquacult. 2014; 26: 271–291. 10.1080/10454438.2014.942124

[13] Rokey GJ, Plattner B, de Souza EM. Feed extrusion process description. R Bras Zootec. 2010; 39: 510–518. 10.1590/S1516-35982010001300055

[14] Pandey B. Pellet technical quality of feeds for Atlantic salmon. Ås, Norway: Norwegian University of Life Sciences 2018 Available from: http://hdl.handle.net/11250/2569989.

[15] Goeritz I, Atorf C, Whalley P, Seymour P, Klein M, Schlechtriem C. Investigation into feed preparation for regulatory fish metabolism studies. J Sci Food Agric. 2014; 94: 438–444. 10.1002/jsfa.6262 23761077

[16] Wold P, Hoehne Reitan K, Cahu C, Zambonino JL, Rainuzzo J, Kjorsvik E. Phospholipids vs. neutral lipids: effects on digestive enzymes in Atlantic cod (*Gadus morhua*) larvae. Aquaculture, 2007; 272: 502–513. 10.1016/j.aquaculture.2007.06.034

[17] Morais S, Conceicao L, Ronnestad I, Koven W, Cahu C, Zambonino JL, Dinis M. Dietary neutral lipid level and source in marine fish larvae: Effects on digestive physiology and food intake. Aquaculture, 2007; 268: 106–122. 10.1016/j.aquaculture.2007.04.033

[18] Hill HA, Trushenski JT, Gause BR, Laporte J. Amending reduced fish meal feeds with phospholipids to improve performance of hybrid striped bass. J Anim Res Nutr. 2015; 1: 1–8. 10.21767/2572-5459.100007

[19] Sheen SS, Chen CT, Ridwanudin A. The effect of partial replacement of fish meal protein by dietary hydrolyzed fish protein concentrate on the growth performance of orange-spotted grouper *Epinephelus coioides*. J Aquac Mar Bio. 2014; 1: 1–6.

[20] Kristinsson HG, Rasco BA. Fish protein hydrolysates: production, biochemical, and functional properties. Crit. Rev. Food Sci. Nutr. 2000; 40; 43–81. 10.1080/10408690091189266 10674201

[21] EFSA Panel on Biological Hazards (BIOHAZ), Ricci A, Allende A, Bolton D, Chemaly M, Davies R, et al Update of the list of QPS‐recommended biological agents intentionally added to food or feed as notified to EFSA 8: suitability of taxonomic units notified to EFSA until March 2018. EFSA Journal. 2018; 16: e05315 10.2903/j.efsa.2018.5315 PMC7009647 32625958

[22] Martínez Cruz P, Ibáñez AL, Monroy Hermosillo OA, Ramírez Saad HC. Use of probiotics in aquaculture. ISRN Microbiol. 2012; 2012: 916845 10.5402/2012/916845 23762761 PMC3671701

[23] Izquierdo M, Koven W. Lipids In: Holt GJ, editor. Larval Fish Nutrition. Hoboken, US: Wiley-Blackwell 2011; p.54. ISBN 978-0-8138-1792-7

[24] Amadou I, Le GW, Shi YH, Gbadamosi OS, Kamara MT, Jin S. Optimized *Lactobacillus plantarum* Lp6 solid‐state fermentation and proteolytic hydrolysis improve some nutritional attributes of soybean protein meal. J Food Biochem. 2011; 35: 1686–1694. 10.1111/j.1745-4514.2010.00493.x

[25] You-Ling G, Cai-Sheng W, Qiu-Hua Z, Guo-Ying Q. Optimization of solid-state fermentation with *Lactobacillus brevis* and *Aspergillus oryzae* for trypsin inhibitor degradation in soybean meal. JIA. 2013; 12: 869–876. 10.1016/S2095-3119(13)60305-6

[26] Hammoumi A, Faid M, El yachioui M, Amarouch H. Characterization of fermented fish waste used in feeding trials with broilers. Process Biochem. 1998; 33: 423–427. 10.1016/S0032-9592(97)00092-7

[27] Ljubobratovic U, Kosanovic D, Vukotic G, Molnar Z, Stanisavljevic N, Ristovic T, et al Supplementation of lactobacilli improves growth, regulates microbiota composition and suppresses skeletal anomalies in juvenile pike-perch (*Sander lucioperca*) reared in recirculating aquaculture system (RAS): A pilot study. Res Vet Sci. 2017; 115: 451–462. 10.1016/j.rvsc.2017.07.018 28777955

[28] Jeyasanta KI, Patterson J. Total lipid, phospholipid and cholesterol contents of six commercially important fishes of Tuticorin, south east coast of India. Sky Journal of Food Science. 2013; 2: 47–53.

[29] Mcsweeney PLH, Fox PF. Chemical methods for the characterization of proteolysis in cheese during ripening. Le Lait, INRA Editions. 1997; 77: 41–76. 10.1051/lait:199713

[30] Higgins BT, Thornton-Dunwoody A, Labavitch JM, VanderGheynst JS. Microplate assay for quantitation of neutral lipids in extracts from microalgae. Anal Biochem. 2014; 465: 81–89. 10.1016/j.ab.2014.07.020 25084552

[31] Cheng YS, Zheng Y, VanderGheynst JS. Rapid quantitative analysis of lipids using a colorimetric method in a microplate format. Lipids. 2011; 46: 95–103. 10.1007/s11745-010-3494-0 21069472

[32] MacWilliams MP, Liao MK. Luria broth (lb) and Luria agar (la) media and their uses protocol. Washington, US: American Society for Microbiology 2006.

[33] Mei C, Yang M, Shu DB, Jiang H, Liu G, Liao Z. Soft sensor based on gaussian process regression and its application in erythromycin fermentation process. Chem Ind Chem Eng. Q.2015; 22: 26 10.2298/CICEQ150125026M

[34] Zhu X, Welling M, Jin F, Lowengrub J. Predicting simulation parameters of biological systems using a Gaussian process model. Stat Anal Data Min. 2012; 5: 509–522. 10.1002/sam.11163 23482410 PMC3589996

[35] Rønnestad I, Thorsen A, Finn RN. Fish larval nutrition: a review of recent advances in the roles of amino acids. Aquaculture. 1998; 177: 201–216.

[36] Rønnestad I, Yúfera M, Ueberschär B, Ribeiro L, Sæle Ø, Boglione C. Feeding behaviour and digestive physiology in larval fish: current knowledge, and gaps and bottlenecks in research. Rev Aquacult. 2013; 5: S59–98. 10.1111/raq.12010

[37] Kolkovski S, Lazo J, Leclercq D, Izquierdo M. Fish larvae nutrition and diet: New developments In: Burnell G, Allan G, editors. New Technologies in Aquaculture: Improving Production Efficiency, Quality and Environmental Management. Sawston, UK: Woodhead Publishing 2009; pp. 315–369. 10.1533/9781845696474.3.315

[38] Kolkovski S. Digestive enzymes in fish larvae and juveniles—implications and applications to formulated diets. Aquaculture, 2001; 200:181–201. 10.1016/S0044-8486(01)00700-1

[39] Kvåle A, Nordgreen A, Tonheim SK, Hamre K. The problem of meeting dietary protein requirements in intensive aquaculture of marine fish larvae, with emphasis on Atlantic halibut (*Hippoglossus hippoglossus L*.). Aquacult Nutr. 2007; 13: 170–185. 10.1111/j.1365-2095.2007.00464.x

[40] Dallagnol AM, Pescuma M, De Valdez GF, Rollán G. Fermentation of quinoa and wheat slurries by *Lactobacillus plantarum* CRL 778: proteolytic activity. Appl Microbiol Biotechnol. 2013; 97: 3129–3140. 10.1007/s00253-012-4520-3 23129182

[41] Di Cagno R, De Angelis M, Lavermicocca P, De Vincenzi M, Giovannini C, Faccia M, Gobbetti M. Proteolysis by sourdough lactic acid bacteria: effects on wheat flour protein fractions and gliadin peptides involved in human cereal intolerance. Appl Environ Microbiol. 2002; 68: 623–633. 10.1128/AEM.68.2.623-633.2002 11823200 PMC126681

[42] Yin Y, Wang J, Yang S, Feng J, Jia F, Zhang C. Protein degradation in wheat sourdough fermentation with *Lactobacillus plantarum* M616. Interdiscip Sci. 2015; 7: 205–210. 10.1007/s12539-015-0262-0 26199213

[43] Addis M. Major causes of meat spoilage and preservation techniques: a review. Food Science and Quality Management. 2015; 41: 101–114.

[44] Tocher DR, Bendiksen EA, Campbell P, Bell JG. The role of phospholipids in nutrition and metabolism of teleost fish. Aquaculture. 2008; 280: 21–34. 10.1016/j.aquaculture.2008.04.034

[45] Sargent JR, Tocher DR, Bell JG. The lipids In: Halver JE, Hardy RW, editors. Fish Nutrition. Cambridge, US: Academic Press 2003; pp. 181–257. 10.1016/B978-012319652-1/50005-7

[46] Mika A, Swiezewska E, Stepnowski P. Polar and neutral lipid composition and fatty acids profile in selected fish meals depending on raw material and grade of products. LWT—Food Sci Technol. 2016; 70: 199–207. 10.1016/j.lwt.2016.02.051

[47] Ruas-Madiedo P, Sánchez B, Hidalgo-Cantabrana C, Margolles A, Laws A. Exopolysaccharides from lactic acid bacteria and bifidobacteria In: Handbook of Animal-Based Fermented Food and Beverage Technology. Hui YH, editor. Florida, US: CRC Press, Taylor & Francis Group 2012; p. 131. ISBN 978143985022049

[48] Gutierrez T, Morris G, Greendoi DH. Yield and physicochemical properties of EPS from *Halomonas* sp. strain TG39 identifies a role for protein and anionic residues (sulfate and phosphate) in emulsification of n‐hexadecane. Biotechnol Bioeng. 2008; 103: 207–216. 10.1002/bit.22218 19160375

[49] Brockerhoff H. Lipolytic Enzymes In: Whitaker JR, editor. Food Related Enzymes. Washington, US: American Chemical Society 1974; pp. 131–145. 10.1021/ba-1974-0136.ch005

[50] Ren X, Zhao X, Turcotte F, Deschênes JS, Tremblay R, Jolicoeur M. Current lipid extraction methods are significantly enhanced adding a water treatment step in *Chlorella* protothecoides. Microb Cell Fact. 2017; 16: 26 10.1186/s12934-017-0633-9 28187768 PMC5303247

[51] Desnuelle P, Savary P. Specificities of lipases. J Lipid Res. 1963;4: 369–384. 14168179

[52] Uppada SR, Akula M, Bhattacharya A, Dutta JR. Immobilized lipase from *Lactobacillus plantarum* in meat degradation and synthesis of flavor esters. J Genet Eng Biotechnol. 2017; 15: 331–334. 10.1016/j.jgeb.2017.07.008 30647671 PMC6296599

[53] Wältermann M, Steinbüchel A. Neutral lipid bodies in prokaryotes: recent insights into structure, formation, and relationship to eukaryotic lipid depots. J Bacteriol. 2005; 187: 3607–3619. 10.1128/JB.187.11.3607-3619.2005 15901682 PMC1112053

[54] Liang K, Zhang Q, Cong W. Enzyme-assisted aqueous extraction of lipid from microalgae. J Agric Food Chem. 2012; 60: 11771–11776. 10.1021/jf302836v 23072503

[55] Shewfelt RL. Fish muscle lipolysis—a review. J Food Biochem. 1981; 5: 79–100. 10.1111/j.1745-4514.1981.tb00663.x

[56] Budzko E. Storage and processing of mackerel—effect on lipid stability [dissertation]. Norway: Norwegian University of Science and Technology; 2018.

[57] Twining CW, Brenna JT, Hairston NG Jr, Flecker AS. Highly unsaturated fatty acids in nature: what we know and what we need to learn. OIKOS. 2016; 125: 749–760. 10.5061/dryad.67dg6

[58] Cahu C, Zambonino Infante J, Takeuchi T. Nutritional components affecting skeletal development in fish larvae. Aquaculture. 2003; 227:245–58. 10.1016/S0044-8486(03)00507-6

[59] Zymon M, Strzetelski J, Pustkowiak H, Sosin E. Effect of freezing and frozen storage on fatty acid profile of calves’ meat. Pol J Food Nutr Sci. 2007; 57:647–50.

[60] Garba L, Ali MSM, Oslan SN, Rahman RNZRBA. Review on fatty acid desaturases and their roles in temperature acclimatisation. J Appl Sci. 2017; 17: 282–295. 10.3923/jas.2017.282.295

[61] Zotta T, Ricciardi A, Ianniello RG, Parente E, Reale A, Rossi F, Iacumin L, Comi G, Coppola R. Assessment of aerobic and respiratory growth in the *Lactobacillus casei* group. PLoS One. 2014; 9: e99189 10.1371/journal.pone.0099189 24918811 PMC4053349

[62] Wójciak KM, Dolatowski ZJ, Kołożyn-Krajewska D, Trząskowska M. The effect of the *Lactobacillus casei* Lock 0900 probiotic strain on the quality of dry-fermented sausage during chilling storage. J Food Qual. 2012; 35: 353–365. 10.1111/j.1745-4557.2012.00458.x

[63] Nayak SK. Probiotics and immunity: A fish perspective. Fish Shellfish Immunol. 2010; 29: 2–14. 10.1016/j.fsi.2010.02.017 20219683

[64] Pandey G. Feed formulation and feeding technology for fishes. IRJP. 2013; 4: 23–30. 10.7897/2230-8407.04306

[65] Lopez-Alvarado J, Kanazaw A. Effect of dietary protein sources in microdiets on feeding behavior and growth of red sea bream, *Pagrus major*, during weaning and metamorphosis. J Appl Aquaculture. 1997; 7: 53–66. 10.1300/J028v07n03_06

[66] Engrola S, Aragão C, Valende LMP, Conceição LEC. Nutritional modulation of marine fish larvae performance In: Emerging issues in fish larvae research. Yúfera M, editor. Switzerland: Springer International Publishing AG 2018; p. 219. ISBN 978-3-319-73244-2

