# Supplementary figures and images for: Solid state treatment with Lactobacillus paracasei subsp. paracasei BGHN14 and Lactobacillus rhamnosus BGT10 improves nutrient bioavailability in granular fish feed
Source: PLoS One. 2019 Jul 11;14(7):e0219558. doi: 10.1371/journal.pone.0219558 (PMC6624013; doi:10.1371/journal.pone.0219558)

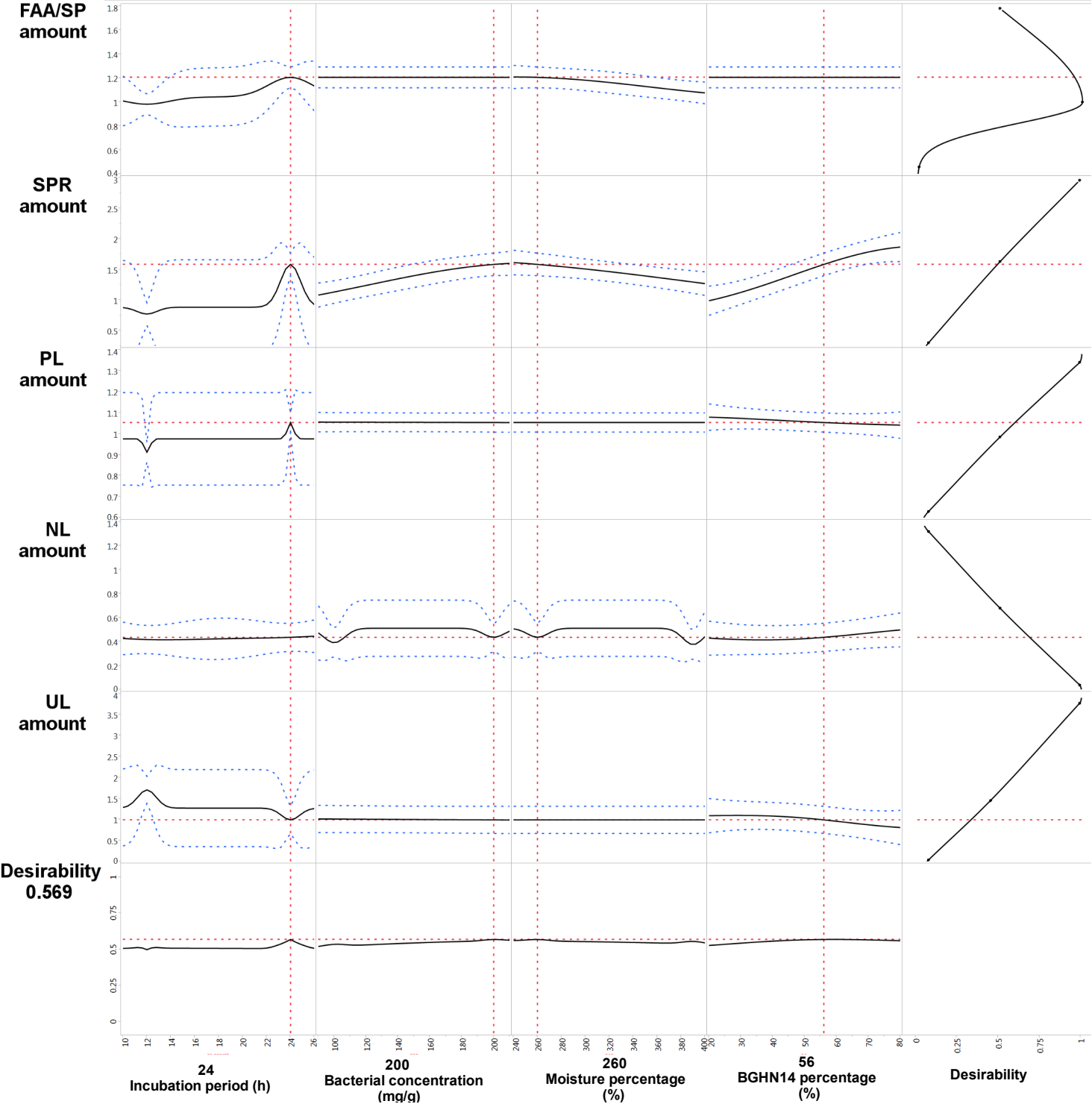

Supplement: S1 Fig — (PDF) [file pone.0219558.s001.pdf]

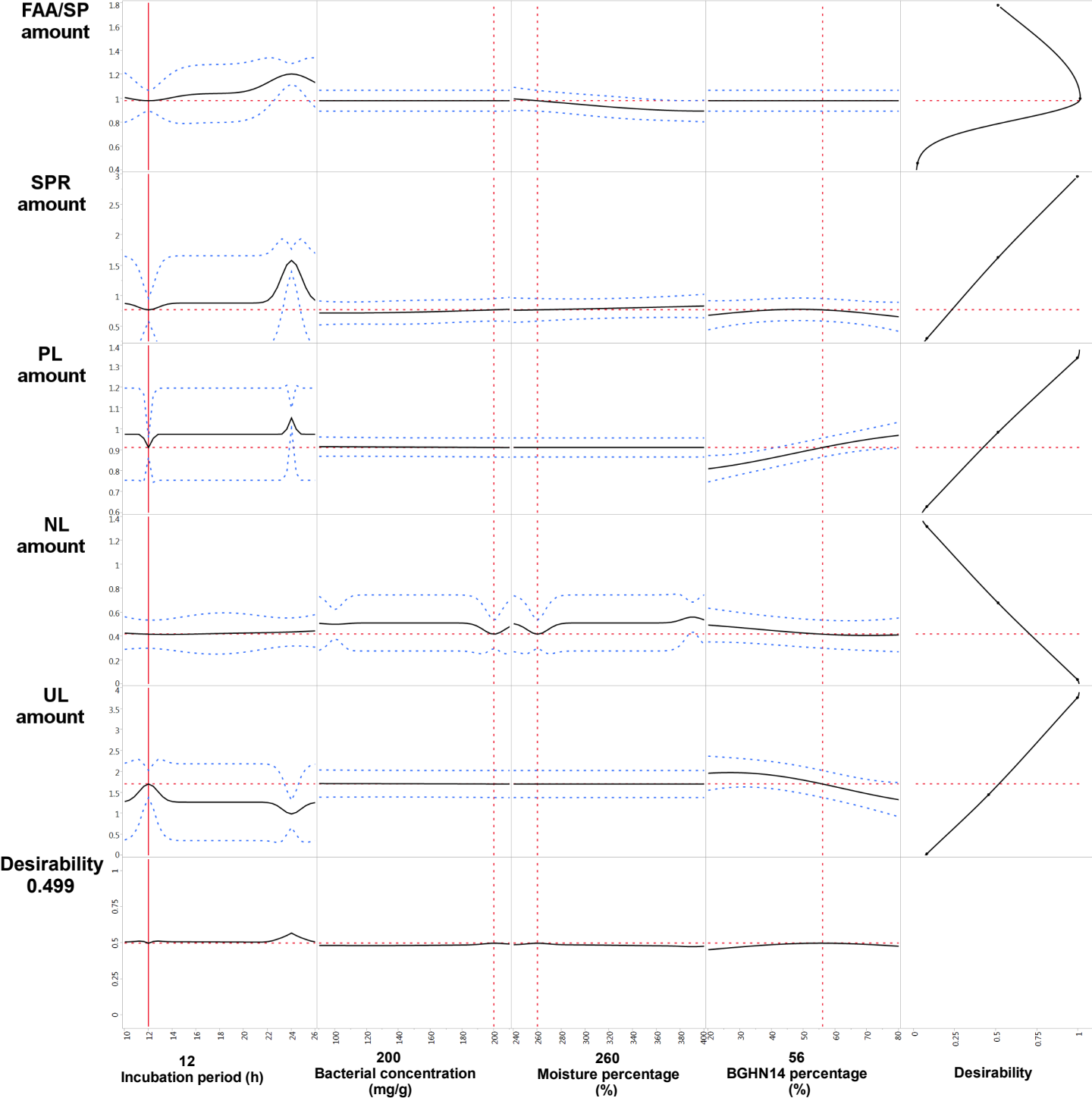

Supplement: S2 Fig — (PDF) [file pone.0219558.s002.pdf]
