# Supplementary material for: Solid state treatment with Lactobacillus paracasei subsp. paracasei BGHN14 and Lactobacillus rhamnosus BGT10 improves nutrient bioavailability in granular fish feed
Source: PLoS One. 2019 Jul 11;14(7):e0219558. doi: 10.1371/journal.pone.0219558 (PMC6624013; doi:10.1371/journal.pone.0219558)
Supplement: S1 Table — Mean values of absolute FAA/SP, SPR, PL, NL and UL amounts in 12 h (a) and 24 h (b) lactobacilli treated and control feed; values are given as μg per mg of dried feed; FAA/SP—free amino acids/short peptides; SPR—soluble proteins; PL—phospholipids; NL—neutral lipids; UL—unsaturated lipids. (PDF) [file pone.0219558.s003.pdf]

S1 Table.

a) 12 h incubation	Mean ± standard error of mean (SEM) (n = 3)				
	FAA/SP amount	SPR amount	PL amount	NL amount	UL amount*
75:25, 260 %, 100 mg g^{-1}	6.29 ± 0.35	0.56 ± 0.06	24.01 ± 2.51	206.16 ± 25.60	3091.89 ± 630.66
50:50, 260 %, 100 mg g^{-1}	4.27 ± 0.89	0.59 ± 0.04	20.90 ± 1.06	188.62 ± 37.07	4144.37 ± 344.75
25:75, 260 %, 100 mg g^{-1}	4.75 ± 0.08	0.41 ± 0.1	18.53 ± 1.42	233.09 ± 11.97	6161.01 ± 1493.35
75:25, 260 %, 200 mg g^{-1}	6.39 ± 0.83	0.38 ± 0.07	22.76 ± 1.39	146.12 ± 23.95	1726.61 ± 187.7
50:50, 260 %, 200 mg g^{-1}	5.50 ± 0.36	0.81 ± 0.14	20.46 ± 0.95	158.89 ± 17.42	4760.16 ± 1487.92
25:75, 260 %, 200 mg g^{-1}	6.92 ± 0.63	0.38 ± 0.03	18.89 ± 1.94	209.52 ± 64.43	3789.87 ± 394.09
260 % control	5.16 ± 0.42	0.73 ± 0.12	21.32 ± 0.14	267.81 ± 19.85	8987.25 ± 397
75:25, 390 %, 100 mg g^{-1}	4.96 ± 0.43	0.62 ± 0.05	24.68 ± 3.85	241.77 ± 42.83	4089.17 ± 921.34
50:50, 390 %, 100 mg g^{-1}	6.20 ± 1.16	0.43 ± 0.14	24.44 ± 3.24	244.37 ± 62.65	5231.20 ± 1617.35
25:75, 390 %, 100 mg g^{-1}	4.42 ± 0.41	0.49 ± 0.12	19.41 ± 0.7	253.75 ± 12.92	4714.77 ± 2046.22
75:25, 390 %, 200 mg g^{-1}	4.75 ± 0.31	0.55± 0.04	20.64 ± 0.25	264.74 ± 51.86	3939.52 ± 767.99
50:50, 390 %, 200 mg g^{-1}	5.01 ± 0.15	0.66 ± 0.13	23.04 ± 1.71	213.64 ± 24.18	4377.44 ± 1305.49
25:75, 390 %, 200 mg g^{-1}	4.78 ± 0.29	0.67 ± 0.13	19.06 ± 2.36	213.72 ± 20.87	5817.55 ± 2045.87
390 % control	4.64 ± 1.15	0.6 ± 0.07	19.25 ± 1.58	278.17 ± 27.91	7142.35 ± 1922.72
Dry control	5.76 ± 0.5	0.76 ± 0.06	24.05 ± 1.85	404.07 ± 59.92	2433.17 ± 838.43

b) 24 h incubation	Mean ± standard error of mean (SEM) (n = 3)				
	FAA/SP amount	SPR amount	PL amount	NL amount	UL amount*
75:25, 260 %, 100 mg g^{-1}	6.45 ± 0.73	0.99 ± 0.06	22.48 ± 1.73	106.96 ± 26.74	2923.84 ± 1673.32
50:50, 260 %, 100 mg g^{-1}	5.73 ± 0.32	0.9 ± 0.06	20.89 ± 0.93	89.99 ± 6.82	6423.52 ± 2222.23
25:75, 260 %, 100 mg g^{-1}	5.57 ± 0.97	0.67 ± 0.07	21.39 ± 0.55	116.21 ± 23.34	4849.71 ± 2400.96
75:25, 260 %, 200 mg g^{-1}	6.15 ± 0.26	1.79 ± 0.32	21.7 ± 0.53	150.89 ± 24.07	2376.75 ± 980.92
50:50, 260 %, 200 mg g^{-1}	5.49 ± 0.55	1.13 ± 0.05	20.61 ± 0.89	89.59 ± 19.05	2206.24 ± 1014.31
25:75, 260 %, 200 mg g^{-1}	6.63 ± 1.13	0.93 ± 0.04	22.21 ± 0.79	115.02 ± 30.22	4811.68 ± 141.21
260 % control	4.8 ± 0.34	0.59 ± 0.11	23.28 ± 0.81	196.88 ± 32.48	6230.93 ± 1258.2
75:25, 390 %, 100 mg g^{-1}	5.03 ± 0.57	0.87 ± 0.02	21.96 ± 0.51	286.14 ± 39.85	3591.15 ± 405.94
50:50, 390 %, 100 mg g^{-1}	6.02 ± 0.65	0.81 ± 0.05	21.2 ± 0.48	145.44 ± 10.63	6635.73 ± 2583.93
25:75, 390 %, 100 mg g^{-1}	5.06 ± 0.36	0.59 ± 0.12	22.25 ± 0.53	144.44 ± 34.47	3657.39 ± 639.66
75:25, 390 %, 200 mg g^{-1}	5.23 ± 0.21	1.21 ± 0.22	19.47 ± 0.65	81.45 ± 38.23	1551.2 ± 956.87
50:50, 390 %, 200 mg g^{-1}	5.79 ± 0.43	0.94 ± 0.09	20.81 ± 0.4	99.27 ± 15.16	5361.23 ± 3553.9
25:75, 390 %, 200 mg g^{-1}	4.25 ± 0.26	0.64 ± 0.14	22.85 ± 0.9	113.74 ± 26.6	2872.32 ± 354.4
390 % control	3.38 ± 0.25	0.32 ± 0.11	25.04 ± 4.5	221.11 ± 15.08	13757.76 ± 7502.47
Dry control	4.85 ± 0.18	0.82 ± 0.15	20.21 ± 0	273.01 ± 18.12	4114.93 ± 323.79

* due to higher amounts of UL present in fishmeal than in coconut oil which was used for UL standard curve preparation, absolute values of UL were higher than the sum of PL and NL.
