# Supplementary material for: Solid state treatment with Lactobacillus paracasei subsp. paracasei BGHN14 and Lactobacillus rhamnosus BGT10 improves nutrient bioavailability in granular fish feed
Source: PLoS One. 2019 Jul 11;14(7):e0219558. doi: 10.1371/journal.pone.0219558 (PMC6624013; doi:10.1371/journal.pone.0219558)
Supplement: S2 Table — Significance values (p) of Shapiro-Wilk test assessment of data normality in different treatment groups; p values higher than 0.05 indicate normal data distribution; FAA/SP—free amino acids/short peptides; SPR—soluble proteins; PL—phospholipids; NL—neutral lipids; UL—unsaturated lipids. (PDF) [file pone.0219558.s004.pdf]

S2 Table.

Treatment	12 h incubation					24 h incubation				
	FAA/SP	SPR	PL	NL	UL	FAA/SP	SPR	PL	NL	UL
75:25, 260 %, 100 mg g^{-1}	0.358	0.59	0.187	0.06	0.673	0.965	0.51	0.503	0.917	0.773
50:50, 260 %, 100 mg g^{-1}	0.736	0.597	0.29	0.179	0.561	0.866	0.703	0.108	0.969	0.102
25:75, 260 %, 100 mg g^{-1}	0.601	0.814	0.845	0.152	0.196	0.778	0.693	0.617	0.281	0.034
75:25, 260 %, 200 mg g^{-1}	0.737	0.263	0.05	0.236	0.559	0.067	0.214	0.454	0.858	0.36
50:50, 260 %, 200 mg g^{-1}	0.571	0.662	0.877	0.261	0.908	0.243	0.246	0.739	0.681	0.058
25:75, 260 %, 200 mg g^{-1}	0.071	0.738	0.963	0.1	0.629	0.851	0.754	0.288	0.828	0.675
260 % control	0.77	0.558	0.22	0.482	0.264	0.022	0.713	0.331	0.989	0.879
75:25, 390 %, 100 mg g^{-1}	0.384	0.233	0.391	0.072	0.547	0.975	0.763	0.364	0.637	0.362
50:50, 390 %, 100 mg g^{-1}	0.154	0.034	0.602	0.21	0.182	0.06	0.818	0.245	0.156	0.101
25:75, 390 %, 100 mg g^{-1}	0.716	0.353	0.195	0.806	0.568	0.483	0.118	0.908	0.996	0.543
75:25, 390 %, 200 mg g^{-1}	0.512	0.14	0	0.814	0.082	0.584	0.258	0.329	0.73	0.238
50:50, 390 %, 200 mg $g-^{1}$	0.936	0.7230	0.698	0.236	0.19	0.808	0.161	0.507	0.923	0.0421
25:75, 390 %, 200 mg g^{-1}	0.18	0.255	0.936	0.9	0.839	0.609	0.511	0.892	0.987	0.247
390 % control	0.475	0.149	0.154	0.472	0.689	0.487	1	0.305	0.949	0.51
Dry control	0.061	0.813	1	0.56	0.724	0.074	0.988	1	0.937	0.163
